# Subchronic Toxicity Study of Oral Anthrafuran on Rabbits

**DOI:** 10.3390/ph14090900

**Published:** 2021-09-04

**Authors:** Michael I. Treshchalin, Helen M. Treshalina, Vasilisa A. Golibrodo, Andrey E. Shchekotikhin, Eleonora R. Pereverzeva

**Affiliations:** 1 Gause Institute of New Antibiotics, 11 B. Pirogovskaya Street, 119021 Moscow, Russia; treshalina@yandex.ru (H.M.T.); vasilisa2006@gmail.com (V.A.G.); shchekotikhin@mail.ru (A.E.S.); pereverzeva-ella@yandex.ru (E.R.P.); 2 Mendeleyev University of Chemical Technology, 9 Miusskaya Square, 125047 Moscow, Russia

**Keywords:** anthrafuran, oral administration, subchronic toxicity, rabbits

## Abstract

A new antitumor multi-target drug anthrafuran, with cellular targets such as topoisomerase I/II and some protein kinases, was obtained in Gause Institute of New Antibiotics and was demonstrated to have a reliable specific effect on different murine and human tumor models by oral administration. In this study, we focused on the evaluation of subchronic toxicity of oral anthrafuran drug formulation (AF) on Chinchilla rabbits. The absence of any changes in the condition or behavior of animals was shown for oral anthrafuran. Changes with reversible and dose-dependent hepato- and nephrotoxicity at low doses, as well as hemato- and gastrointestinal toxicity at high doses, were confirmed pathomorphologically. The identified toxic properties are extremely valuable, since oral anthrafuran does not have the limiting cardio- and myelotoxicity. Anthrafuran with 2 mg/kg/day or 6 mg/kg/day doses was administrated orally over 15 days. Investigations include assessment of the body weight, hematological and serum biochemical parameters and urinalysis, electrocardiography and pathomorphological evaluation of the internal organs. Quantitative data were processed statistically with Student’s *t*-Test, *p* < 0.05. Revealed during the subchronic study were the favorable toxicological properties of oral anthrafuran as opposed to clinical anthracyclines, oral idarubicin, or parenteral doxorubicin, which allows it to be considered promising for further research.

## 1. Introduction

Heteroarenanthracenediones containing cyclic diamines in the side chain are known to have high antiproliferative activity [1,2,3,4,5]. Previously described multi-targeting (*S*)-3-[(3-amino-1-pyrrolidinyl)carbonyl]-4,11-dihydroxy-2-methylantra[2,3-*b*]furan-5,10-dione (hereinafter anthrafuran) is capable of simultaneously inhibiting topoisomerase 1 and 2, as well as protein kinases, and induces the death of tumor cells of various histogenesis [6,7]. An investigation of the anthrafuran anticancer properties in vivo showed a high efficacy with a wide dosage diapason against transplantable tumors by intraperitoneal administration [3]. A study of the kinetics of dissolution of anthrafuran substance in bio-relevant media (for example, in artificial gastric juice at 37 °C) showed that anthrafuran refers to preparations with a low biopharmaceutical solubility (look for Biopharmaceutics Classification System, BCS) [8]. To increase biopharmaceutical solubility, an oral dosage form based on anthrafuran and liquid pharmaceutically acceptable carriers with a high release rate of the active ingredient are obtained [9]. Most anticancer drugs are applied parenterally, as this route of administration provides fast and maximal bioavailability of the agents and therefore, accurate dosing during the whole course of chemotherapy. Novel orally administrated agents have a well-delivered formulation to achieve adequate bioavailability. High bioavailability makes it possible to achieve complete or partial remission with oral administration of certain alkylating agents, antitumor antibiotics, and other classes of anticancer drugs. In this regard, more and more oncologists have begun to give preference to oral chemotherapy drugs, since this route of administration improves the quality of life of patients and reduces their time in the clinic [10]. Thereby, the searches for novel orally bioavailable agents are real directions in anticancer drug development. Earlier, it was revealed that moderately subchronic toxicity on the rodents (rats) by the oral route of administration [11,12].

The aim of the present study was to investigate the toxicological properties of oral anthrafuran in rabbits.

## 2. Results

### 2.1. Functional Indicators and 0bservations

Administration of anthrafuran formulation did not cause mortality in any of the treated groups. No signs of dyspepsia, abnormal behavioral reactions or neurological disorders were observed.

### 2.2. Dynamics of Body Weight

**Figure 1:**
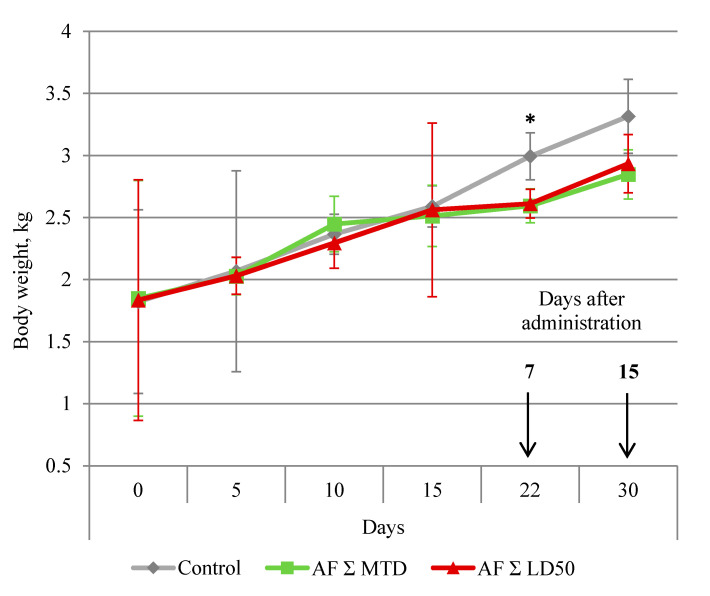
Dynamic of rabbit’s body weight during and after oral administration of anthrafuran formulation (AF). Note: * Values are significantly different to the control, *p* ≤ 0.05, *n* = 6.

The dynamics of the body weight changes are presented in Figure 1. By day 7 after the treatment (22th experiment day), all male rabbits who obtained anthrafuran were losing weight. By day 15 post-treatment (30th experiment day), the body weight in both treated groups did not differ from the control.

### 2.3. Hematological Tests

Peripheral blood tests did not reveal any changes in the quantity of the total and differential leukocyte count, erythrocytes and thrombocytes count, the value of hemoglobin, or hematocrit.

**Figure 2:**
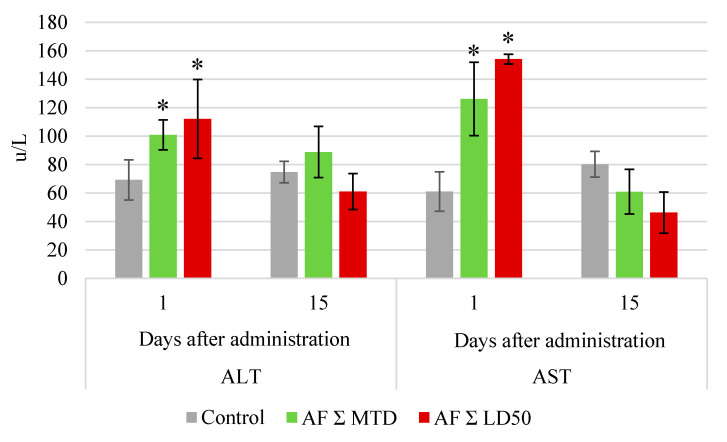
Serum alanine aminotransferase (ALT) and aspartate aminotransferase (AST) levels in rabbits after the end of oral anthrafuran formulation (AF) administration course. Note: * Values significantly different from control, *p* ≤ 0.05, *n* = 6.

Biochemical serum tests showed an increase in the alanine aminotransferase (ALT) and aspartate aminotransferase (AST) levels in all groups immediately after the end of drug administration course (Figure 2). By the end of the experiment, this parameter was similar to control.

### 2.4. Heart Functions

On day 1 as well as on day 15 post treatment, the electrocardiographic (ECG) parameters in both groups were similar to control.

### 2.5. Internal Organs

The data on the drug influence on WI are presented in Table 1. In the group which obtained a high dose of anthrafuran, by day 1 the thymus weight was decreased and liver weight was increased as compared to control. By day 15, WI of the internal organs in all groups which obtained anthrafuran were similar to control.

### 2.6. Urine Analysis

The clinical urine analysis on day 1 post-treatment showed an increase of urobilinogen, ketones, and protein and a decrease of the specific gravity of urine in both tested groups (Table 2). By the end of the observation, the results of urine tests of the experimental animals did not differ from the control.

### 2.7. Histological Evaluation

**Figure 3:**
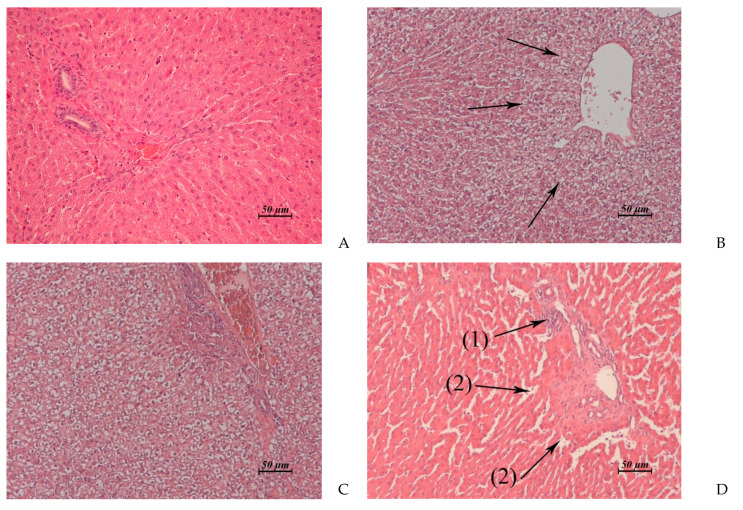
(**A**–**D**). Rabbit liver. (**A**) Untreated control. (**B**) AF, 15 × 2 mg/kg/day, 1 day post treatment. Vacuolar dystrophy of hepatocytes around of the central vein. (**C**) AF, 15 × 6 mg/kg/day, 1 day post treatment. Total pronounced vacuolar dystrophy of hepatocytes. (**D**) AF, 15 × 6 mg/kg/day, 15 day post treatment. Edema around the triad. Small micronecrotic focus in the vicinity of the triad (1). Hyperplasia of hepatobiliary ducts and enlargement of connective tissue around them. (2) ×20.

**Figure 4:**
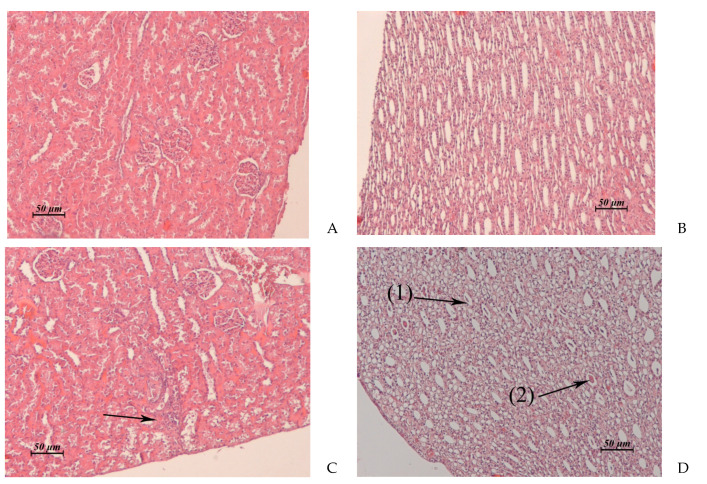
(**A**–**D**). Rabbit kidney. (**A**) Untreated control. Cortical zone. (**B**) Untreated control. Medullary zone. (**C**) AF, 15 × 2 mg/kg/day, 1 day post treatment. Foci of destruction of the convoluted tubules in renal cortex. (**D**) AF, 15 × 2 mg/kg/day, 1 day post treatment. Cellular detritus (1) and hyaline cylinders (2) in the lumen of some medullary tubules. ×20.

**Figure 5:**
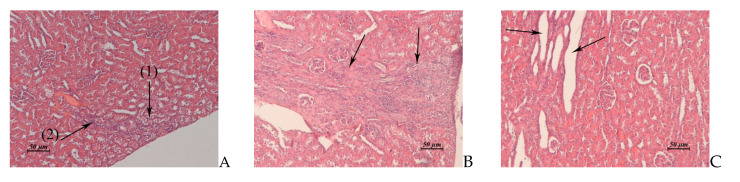
(**A**–**C**). Rabbit kidney. (**A**) AF, 15 × 6 mg/kg/day, 1 day post treatment. Cortical zone. Foci of vacuolar dystrophy (1) and destruction in the epithelial layer (2) of the convoluted tubules. (**B**) AF, 15 × 6 mg/kg/day, 15 day post treatment. Fibrosis of some necrotic nephrons passing through cortical and juxtamedullary zones. (**C**) AF, 15 × 6 mg/kg/day, 15 days post treatment. Juxtamedullary zone. Cysts lined with squamous epithelium at the site of destructed convoluted and medullary tubules. ×20.

**Figure 6:**
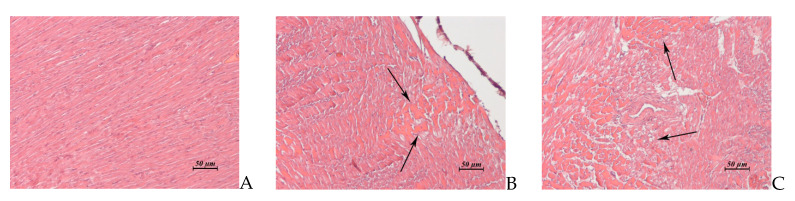
(**A**–**C**). Rabbit myocardium. (**A**) Untreated control. (**B**) AF, 15 × 2 mg/kg/day, 1 day post treatment. Foci of toxic cardiomyopathy. (**C**) AF, 15 × 6 mg/kg/day, 15 day post treatment. Extensive foci of interstitial edema associated with toxic cardiomyopathy. ×20.

The results of the pathomorphological study of the inner organs after drug administration course with anthrafuran formulation are presented in Table 3 and in Figure 3, Figure 4, Figure 5 and Figure 6.

No pathological changes were observed in other organs.

## 3. Discussion

Anthracyclines and structurally similar anthracenediones, which are highly effective antineoplastic agents widely used for the treatment of malignant tumors. Anthrafuran, a heterocyclic derivative of anthracenedione, demonstrated a reliable antitumor effect on different murine tumor models for oral administration [7]. The design of the subchronic toxicity study on rabbits—15 daily oral administrations at two dose levels of 2 mg/kg/day (1/15 MTD) and 6 mg/kg/day (1/15 LD_50_)—followed by a 15-day observation period, allowed us to identify all of the main toxic effects of anthrafuran. The study showed that AF has a more favorable toxicological profile compared to doxorubicin and other anthracyclines. Signs of doxorubicin toxicity observed in the chronic toxicity study, such as a reduction of body weight, cardiotoxicity, depression of hematopoiesis and spermatogenesis, gastrointestinal tract (GIT), and renal damage, are well known and have been described by many authors [13,14,15].

AF, independent of the dose, when administered daily, did not affect the consumption of food and water and did not cause the death of animals. AF induced a short-term reduction of the body weight, which could be interpreted as indirect evidence of gastrointestinal toxicity. No other signs of gastrointestinal toxicity were detected either by clinical or morphological methods.

The major dose-limiting toxicities of doxorubicin, as well as daunorubicin, epirubicin, and idarubicin, are neutropenia and cardiomyopathy [15]. Most researchers note that the hematological toxicity of anthracyclines is equivalent to their cardiotoxicity [16]. When using anthrafuran, hematological parameters remained within the physiological norm throughout the study. A decrease in WI of thymus in animals treated with a high dose of anthrafuran may indicate a possible manifestation of hematological toxic properties during overdose. The pathomorphological study confirmed that administration of AF at the dosage level of 6 mg/kg/day causes moderate atrophy of lymphoid tissue of the thymus, spleen, and lymph nodes. The structure of the spleen and the lymph nodes is fully restored. In individual lobules of the thymus it persists for two weeks.

Signs of cardiotoxicity were detected only by pathomorphological examination. After administration of AF at the dosage level of total MTD, the morphological features of toxic cardiomyopathy were moderately expressed, and by the end of the observation, the myocardial structure was completely restored. A high dose of anthrafuran caused the same changes as a low one on day 1 after the course, but after day 15 post treatment, they intensified. It can be assumed that the drug, when the tolerated doses are exceeded, is able to display cardiotoxic properties.

Clinical, laboratory, and morphological investigations have demonstrated that AF applied in both doses studied exhibits hepato- and nephrotoxic properties. Hepatotoxicity was manifested in an increase of transaminases levels in the blood serum of rabbits and was enhanced in the mass coefficient of the liver. Lesions of the liver structure were expressed moderately after a low dose administration of anthrafuran formulation on day 1 post treatment and 2 weeks later, liver morphology was similar to control. When applying anthrafuran in a dose totaling LD_50_, the structure of the liver by the end of the experiment was restored only partially.

Signs of nephrotoxicity were manifested in an increase in the level of protein and urobilinogen in urine and a decrease in its specific gravity. The degree of failure of kidney morphology and the rate of recovery depended on the value of the applied dose. A high dose of anthrafuran caused necrosis in the cortical and medullary regions of the kidney. The damaged areas have undergone fibrosis.

It is well known that doxorubicin induces considerable testicular toxicity in men and animals [17,18]. Importantly, no pathological changes after AF administration were observed in the rabbit testis.

Toxic properties of oral anthrafuran revealed in this study correspond to those previously detected in rats [11]. Some differences in the results of the investigations, apparently, are associated with the fact that chronic toxicity in rats was examined using the drug substance, and in the study on rabbits, a drug formulation was used. In previous pharmacokinetic researches, it was found that after oral administration of the drug formulation, AF was absorbed faster, and its maximum concentration in the blood was higher than after the administration of a substance [9]. AF substance is absorbed 2 times slower from the gastrointestinal tract into the blood; therefore, its damaging effect on the gastric and intestinal mucosa of rats is more prolonged and is manifested to a greater extent than in rabbits. A longer circulation of the drug substance in the blood can also explain the more pronounced hematological toxicity of AF on rats.

Altogether, these results provide evidence that toxicity of oral anthrafuran in both cases depended on dose, and multiple administration of therapeutic doses of the drug produced transient completely reversible toxic effects.

## 4. Materials and Methods

**Figure 7:**
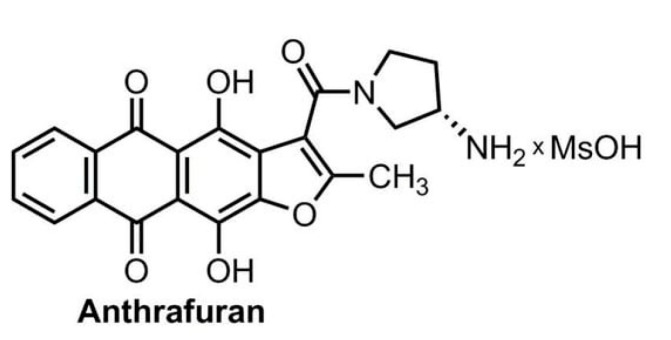
Anthrafuran chemical structure.

A substance of anthrafuran (Figure 7), purity 99%, (*S*)-3-(3-aminopyrrolidine-1-carbonyl)-4,11-dyhydroxy-2-methylanthra[2,3-*b*]furan-5,10-dione methanesulfonate dyhydrate was synthesized following the previously reported method [2,3,6].

All experiments in vivo were performed in accordance with the European Convention for the Protection of Vertebrate Animals [19], and the National standard of the Russian Federation R 53434-2009 «Good Laboratory Practice» [20].

The study was carried out on adult male Chinchilla rabbits (1.8–2.0 kg) obtained from the animal breeding unit of the FMBA (Scientific center for biomedical technologies of the Federal biomedical agency, Moscow, Russia). The animals were randomly divided into groups (*n* = 6). The animal study was approved by the Ethics of Animal Experimentation of Gause Institute of New Antibiotics (protocol No 11/2020).

The treatment regimen was 15 × 2 mg/kg and 15 × 6 mg/kg, with a 24-h interval between administrations. Total doses were equivalent to the maximum tolerated dose (MTD) or a medium lethal dose (LD_50_) of anthrafuran, respectively. Doses were calculated from appropriate doses for rats established in the study of acute toxicity of the drug, using the dose conversion factors [11,21]. Untreated animals were used as a control.

Anthrafuran formulation (AF) were in the form of gelatin capsules prepared individually for each animal, based on its body weight. Capsules were placed on the root of the tongue and 10 mL of water was introduced into the oral cavity using a soft silicone probe plastic syringe. Dosage preparation for administration was carried out once a week according to a change in the weight of the test animals. Doses before administration were stored at room temperature, but not higher than 25 °C.

In the course of the observation periods, the animals were followed up for any adverse effects. Body weight and food consumption were evaluated weekly. The hematological tests were performed using an automated hematology analyzer (Abacus Junior Vet, Diatron, Budapest, Hungary). Blood was withdrawn from the marginal ear vein in healthy animals before drug administration, then during the drug administration course (days 0, 7, 15), and after the end of the drug administration course (days 3, 7, 15). The following parameters were checked: leukocyte count with differentiation type, erythrocyte count, hemoglobin, thrombocyte count, and hematocrit.

The following clinical biochemistry parameters were determined automatically using a biochemical analyzer (ChemWell, Awareness Technology, Inc., Palm City, Florida, USA) on days 1 and 15 post-treatment: alanine aminotransferase (ALT), aspartate aminotransferase (AST), alkaline phosphatase, creatinine, glucose, serum urea, bilirubin total and direct, total protein, and albumin.

The urinalysis tests were performed on days 1 and 15 post-treatment automatically using a Laura Smart analyser (Lachema, Prague, Czech Republic).

Electrocardiographic examination (second standard lead) was performed on days 1 and 15 post-treatment (electrocardiograph АКSION EK1Е-07, Moscow, Russia).

The animals were euthanized on days 1 and 15 after the end of drug administration course. Necropsy was performed for the all animals. Thoracic and abdominal cavities and internal organs were inspected macroscopically. The organs were fixed in 10% neutral formalin, embedded in paraffin, and cut. The sections 5 µ thick were stained with hematoxylin-eosin.

Statistical analysis was performed using the Student’s *t*-Test. Mean values and standard deviations were calculated for body weights, hematological parameters, and relative organ weights. The difference between the groups was considered significant at *p* ≤ 0.05.

## 5. Conclusions

Thus, the evaluation of subchronic toxicity of AF on the rabbits demonstrated less toxicity for oral administration compared to doxorubicin and other anthracyclines and anthracenediones. Most of its side effects are reversible within 15 days. Overall, the results of the studies indicate the high potential for the further development of this novel anticancer drug.

## Figures and Tables

**Table 1:** Weight indices of internal organs of rabbits treated with anthrafuran orally.

Groups	Thymus	Heart	Spleen	Kidney	Liver
Day 1 post treatment					
AF, Σ MTD	0.16 ± 0.08	0.33 ± 0.09	0.06 ± 0.02	0.34 ± 0.09	3.90 ± 0.77
AF, Σ LD_50_	* 0.14 ± 0.03	0.24 ± 0.01	0.05 ± 0.01	0.26 ± 0.03	* 7.53 ± 1.20
Control	0.21 ± 0.02	0.24 ± 0.01	0.07 ± 0.02	0.28 ± 0.02	3.67 ± 1.34
Day 15 post treatment					
AF, Σ MTD	0.16 ± 0.04	0.23 ± 0.03	0.05 ± 0.01	0.26 ± 0.02	2.47 ± 0.47
AF, Σ LD_50_	0.16 ± 0.09	0.24 ± 0.01	0.04 ± 0.02	0.25 ± 0.02	2.61 ± 0.17
Control	0.15 ± 0.05	0.26 ± 0.05	0.06 ± 0.01	0.24 ± 0.01	2.44 ± 0.22

Note: * Values significantly different from control, *p* ≤ 0.05. In the each group *n* = 6.

**Table 2:** Clinical urine analysis of rabbits treated with anthrafuran orally.

Groups	Urobilinogen (μmol/L)	Ketones (mmol/L)	рН	Protein (g/L)	Specific Weight
Day 1 post treatment					
AF, Σ MTD	* 17.0	* 0–0.5	6.6	* 5	* 1.00
AF, Σ LD_50_	* 17.0	* 0–0.5	6.5	* 5	* 1.00
Control	negative	negative	6.6	≤0.33	1.02
Day 15 post treatment					
AF, Σ MTD	negative	negative	6.3 ± 0.3	0.53 ± 0.4	1.020 ± 0.003
AF, Σ LD_50_	negative	negative	6.5 ± 0.5	0.53 ± 0.4	1.010 ± 0.01
Control	negative	negative	6.5	≤0.33	1.015 ± 0.005

Note: * Values significantly different from control, *p* ≤ 0.05, *n* = 6.

**Table 3:** Pathomorphological findings after administration of anthrafuran formulation (AF) on days 1 and 15 post treatment course.

Organ	Day 1st		Day 15th	
	Anthrafuran of 2 mg/kg/day	Anthrafuran of 6 mg/kg/day	Anthrafuran of 2 mg/kg/day	Anthrafuran of 6 mg/kg/day
Liver (Control: Figure 3A)	Vacuolar dystrophy of hepatocytes around of the central veins (Figure 3B). Single small foci of micro necrosis in the vicinity of triads. Moderate lympho-histiocytic infiltrates around the hepatobiliary ducts	Total pronounced vacuolar dystrophy of hepatocytes (Figure 3C). Micronecrotic foci of different sizes in the vicinity of triads and central veins	Similar to control	Vacuolar dystrophy of hepatocytes around of some central veins. Moderate edema around the triads. Rare small micro necrotic foci in the vicinity of the triads. Hyperplasia of hepatobiliary ducts and enlargement of connective tissue around them, sometimes fibrosis around the triads (Figure 3D).
Kidney (Control: Figure 4A, B)	Cortical zone: single foci of vacuolar dystrophy and destruction of the convoluted tubules (Figure 4C). Medullary zone: cellular detritus and rare hyaline cylinders in the lumen of some medullary tubules (Figure 4D)	Cortical and medullary zones: severe perivascular edema. Cortical, juxtamedullary and medullary zones: multiple small necrotic or destructed foci in the epithelial layer of the convoluted and medullary tubules (Figure 5A)	Similar to control	Fibrosis of some necrotic nephrons passing through cortical, juxtamedullary and medullary zones (Figure 5B). Cysts lined with squamous epithelium at the site of destructed convoluted and medullary tubules (Figure 5C). Thickening of the glomerular capsule and focal atrophy of the glomerular capillary network in the glomeruli adjacent to the fibrosis area.
Myocardium (Control: Figure 6A)	Single small foci of toxic cardiomyopathy (Figure 6B)	Two animals exhibited multiple small foci of toxic cardiomyopathy	Similar to control	Multiple extensive foci of interstitial edema associated with toxic cardiomyopathy (Figure 6C).
Spleen	Similar to control	Similar to control	Moderate atrophy of lymphoid tissue of some follicles	Large germinal centers in individual follicles.
Thymus	Moderate atrophy of lymphoid tissue in the medullary zone of some lobules	Moderate atrophy of lymphoid tissue in the medullary zone of lobules	Similar to control	Moderate atrophy of lymphoid tissue in the medullary zone of lobules.
Lymphatic node	Similar to control	Moderate atrophy of lymphoid tissue of some lymphoid follicles	Similar to control	Similar to control.

## Data Availability

Data are contained within the article.
